# Mining Prognosis Index of Brain Metastases Using Artificial Intelligence

**DOI:** 10.3390/cancers11081140

**Published:** 2019-08-09

**Authors:** Shigao Huang, Jie Yang, Simon Fong, Qi Zhao

**Affiliations:** 1 Cancer Center, Institute of Translational Medicine, Faculty of Health Sciences, University of Macau, Taipa 999078, China; 2 Department of Computer and Information Science, University of Macau, Taipa 999078, China; 3 Department of Electromechanical Engineering, Chongqing Industry&Trade Polytechnic, Chongqing 408000, China; 4 Center of Medical Instruments, Zhuhai Institute of Advanced Technology Chinese Academy of Sciences, Zhuhai 519000, China

**Keywords:** brain metastases, radiosurgery, prognosis index, artificial intelligence

## Abstract

This study is to identify the optimum prognosis index for brain metastases by machine learning. Seven hundred cancer patients with brain metastases were enrolled and divided into 446 training and 254 testing cohorts. Seven features and seven prediction methods were selected to evaluate the performance of cancer prognosis for each patient. We used mutual information and rough set with particle swarm optimization (MIRSPSO) methods to predict patient’s prognosis with the highest accuracy at area under the curve (AUC) = 0.978 ± 0.06. The improvement by MIRSPSO in terms of AUC was at 1.72%, 1.29%, and 1.83% higher than that of the traditional statistical method, sequential feature selection (SFS), mutual information with particle swarm optimization(MIPSO), and mutual information with sequential feature selection (MISFS), respectively. Furthermore, the clinical performance of the best prognosis was superior to conventional statistic method in accuracy, sensitivity, and specificity. In conclusion, identifying optimal machine-learning methods for the prediction of overall survival in brain metastases is essential for clinical applications. The accuracy rate by machine-learning is far higher than that of conventional statistic methods.

## 1. Introduction

The prognosis for patients with brain metastases (BM) is known to be poor, as BM is one of the most deadly among various types of cancers [1,2,3,4]. Ranging from early detection to intervention therapy, many innovative management models have been formulated with the goal of lowering the fatality rate of BM. Nevertheless, in the clinical practices, exploring the prognosis index markers of patients is often difficult and costly. Meanwhile, the challenging task of brain metastases survivability prediction could strongly benefit from the development of personalized and precise medicine. In this context, artificial intelligence technology can be used to predict cancer as a means of inexpensive and practical research methodology.

It is forecasted that big data and bioinformatic technology will stay prevalent in the coming year [5,6,7,8,9]. Artificial intelligence (AI) has been used to diagnose and classify cancer for more than two decades. In contrast, utilizing bioinformatic methods to seek the prognosis index is a relatively new approach [10]. Prognostic indices are commonly used in the field of brain metastases radiotherapy for guiding patient decision-making and clinical trial stratification. Cancer prognosis is lately becoming one of the most important research areas, having profound significance in cancer precision treatment [11,12,13]. With the development of artificial intelligence technology [14,15,16], machine learning has been applied on prognosis of breast cancer [17], lung cancer [18], among others. But only a few studies have investigated their relevance in BM prognosis [19]. In Hosny’s perspective opinion, they compared artificial versus human intelligence and established a comprehensive understanding of AI, with a general focus on applications in cancer to have an outlook of the AI method [20]. Chang et al. [21] reported that the prognosis validate by machine learning is superior with the best accuracy (accuracy = 93.81%; AUC = 0.90) for oral cancer prognosis. Therefore, a variety of prognostic indices have been established to evaluate the prognosis of patients with brain metastases. Subsequently, clinicians can use them as a guide for treatment decision making and for trial eligibility. To date, commonly used clinical prognosis indices in BM patients included Recursive Partitioning Analysis (RPA), Graded Prognostic Assessment (GPA), Score Index for Radiosurgery (SIR), and Basic Score for Brain Metastases (BSBM). A previous study concluded that BSBM is the most accurate index for the prediction of patients’6- and 12-months overall survival [22]. However, there have seldom been any research study focusing on the mining of prognosis index markers through attribute reduction and classification methods, especially based on particle swarm optimization technology [23]. Attribute reduction is useful, as the association between the prognosis features and primary tumor type, metastasis, and other clinical outcomes, are proven [24,25,26].

In this study, an improved algorithm called MIRSPSO (mutual information and rough set of particle swarm optimization) is proposed to select the most optimized prognostic indices from all the prognostic indices. MIRSPSO identifies optimal machine learning methods for the most accurate prediction of overall survival in brain metastases. Machine learning is also used to conclude that the prognosis index and the clinical performance of the best prognosis was superior to conventional statistic methods in terms of accuracy, sensitivity, and specificity. Overall, it shows that the use of an AI method as a feasible and convenient application that seeks an optimized prognosis index for BM patients in clinical use is possible.

## 2. Results

### 2.1. Clinical Characteristics and Overall Survival

**Figure 1:**
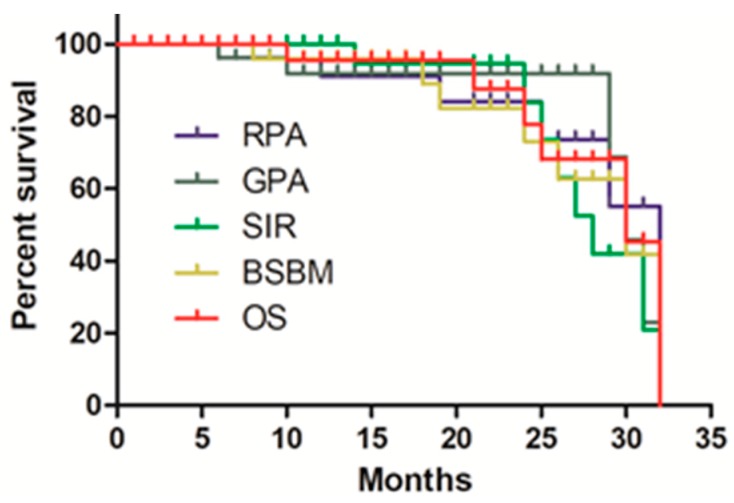
Overall survival curve for the four Prognostic Indices. RPA, Recursive Partitioning Analysis; GPA, Graded Prognostic Assessment; SIR, Score Index for Radiosurgery; BSBM, Basic Score for Brain Metastases; and OS, overall survival curve for all patients.

The median survival time (MST) was 24 months (95% CI 6.708–9.292 months). The 1- and 2-year survival rates were 80% and 42%, respectively. The survival results classified by prognostic indices are shown in Figure 1. BSBM is the most accurate prognosis to predict patients’ chances of 1-and 2-year overall survival.

### 2.2. Qualitative Feature Analysis

The seven prominent features are abbreviated and tabulated in Table 1. We proposed a mutual information particle swarm attribute reduction method based on sequence features to explore the relationship between benign and malignant tumors of cancer patients and each marker. MIRSPSO that is based on meta-heuristic mutual information is compared with the traditional statistical method (SFS), the rough set attribute reduction method (MISFS), the attribute reduction method based on particle swarm optimization, and the rough set attribute selection method.

As shown in Table 2, the seven features which are abbreviated in alphabets form different sequences. The positions of the features in a sequence are sorted from left to right by their descending importance. SFS ranks feature C, “If chemical therapy” as most important; feature N, “Number of lesions” is ranked as least important, located at the end of the sequence. Similarly, MISFS ranks “Age” as the most important feature in the top position and “Max lesion volume” is least important at the end. However, SFS and MISFS only sort the order of the features, without filtering and removing non-important features. As such, the presence of the unimportant features is likely to cause interference in the induction of the classification model. Therefore, our proposed method first extracts the core attribute(s) through the attribute reduction technique. At the same time, it weighs the dependence of decision attributes and the mutual information between attributes. The result of filtering and sorting are shown as PSO-SFS and MIRSPSO in Table 2. We can see that only 4 or 5 most important features are selected into a subset as core attributes through attribute reduction. MISFS only considers the attribute dependence, selects five features, P, E, A, C, K as core attributes, while MIRSPSO picks P, A, E, C four features as core attributes. More core attributes are retained when compared with the attribute reduction method used in the BSBM prognosis index. Consistently, the feature prognosis index of primary tumor control (P), which is calculated by MIRSPSO method, matches with the previous clinical result [22].

**Figure 2:**
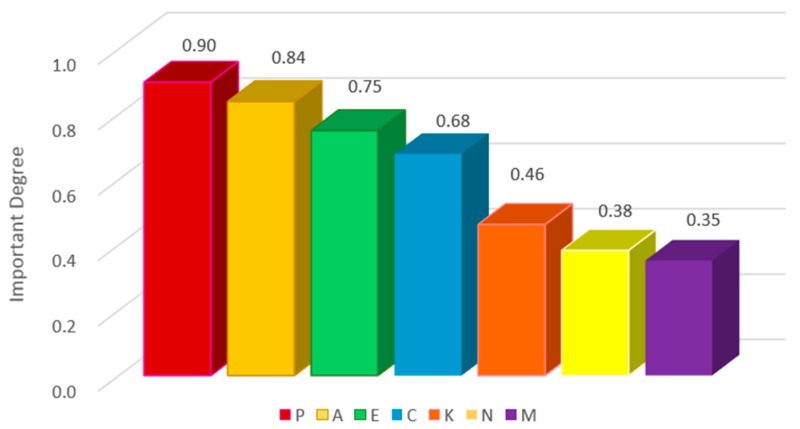
Distribution of degrees of importance for different features in patients.

By considering both attribute dependence and mutual information between attributes, two different weights could be set as two individual factors contributing to the outcomes. A comparison of attribute reduction and sorting under different ω weights is shown in Table 3. Three attributes are reduced for each of the 9 groups of weights, and 4 features (P, A, E, C) are selected as core attributes, indicating that the different distribution of weights does not affect the number of attributes reduced. Different weight distributions will affect the ranking of feature importance, but the ranking order of the features is basically stable. For example, the weights of 0.7 and 0.3 are similar to that of 0.6 and 0.4. The difference is that E and C are out of the original order. We can see that in the leftmost (most important) feature, the probabilities of P, A, E and C ranking that ranks first, second, third, and fourth are88.89%, 66.67%, 55.56%, and 66.67%, respectively. This indicates that P(Primary control) has the highest degree of dependence and mutual information among all the attributes, as shown in Figure 2. Considering attribute dependence and mutual information together can improve the effectiveness and stability of the reduction results. The relationship between cancer tumor features and patients are fully explored, thus providing a more powerful guarantee for the identification and decision making of benign or malignant cancer tumors.

**Figure 3:**
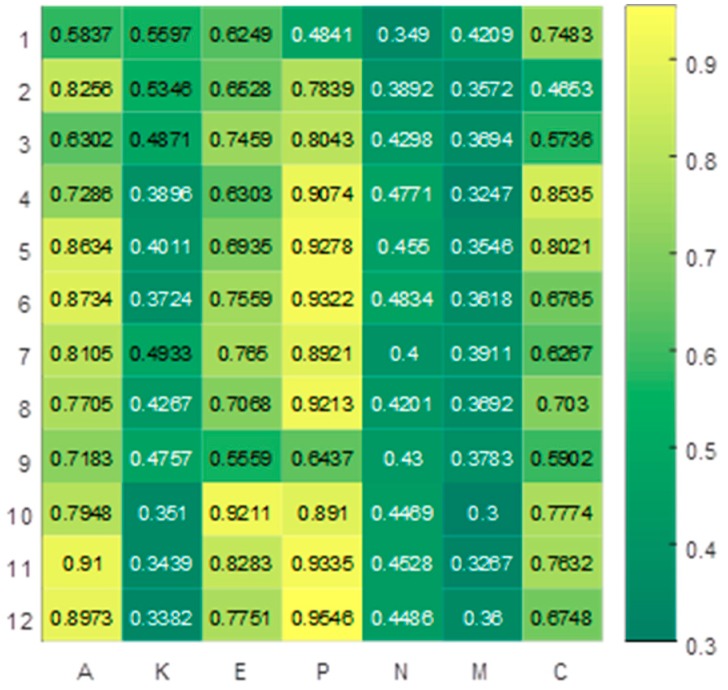
Heat map illustrating the predictive performance (AUC) of the relationship between cancer features and importance degree in patients. P: Primary tumor control was the most important degree in patients.

Primary tumor control wasmost important in patients. The levels of significance for each feature are visualized in the form of a heat map in Figure 3. The abscissa is the seven features obtained, and the ordinate is the different experimental methods. For the ordinate, 1–3 are SFS, MISFS, and MIPSO, respectively, and 4–12 are the importance distribution obtained by the MIRSPSO method under different weights. A and P are mostly close to yellow, K, N, and M are mostly close to green, and E and C are mostly close to the color between yellow and green. The distribution of importance of each feature can be clearly seen by referring to the color bar of significance on the right.

### 2.3. Feature Selection and Prognosis Prediction

**Figure 4:**
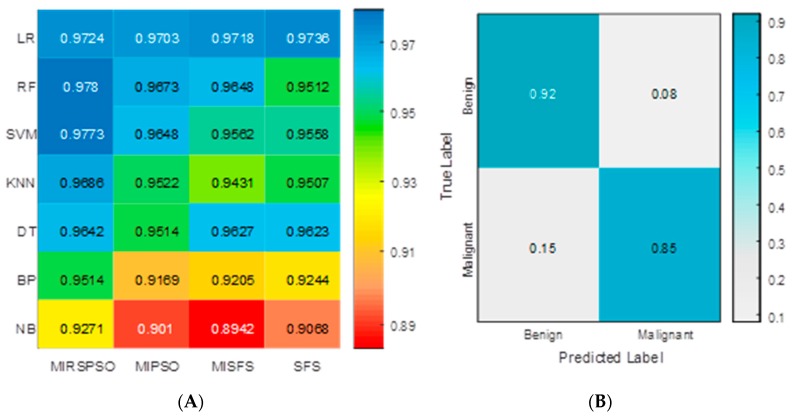
Predictive performance of the constructed classifier in the test cohort. (**A**) Heatmap depicting the prognostic performance (AUC) of feature selection (in rows) and classification (in columns) methods. It depicts the mean AUC of all the crossed methods, along with standard deviation (SD) and the 0.25 and 0.75 (the quantile values). It can be observed that MIRSPSO+RF, MIRSPSO+SVM had the highest values of AUC. (**B**) Confusion matrix with the MIRSPSO+RF classifier.

**Figure 5:**
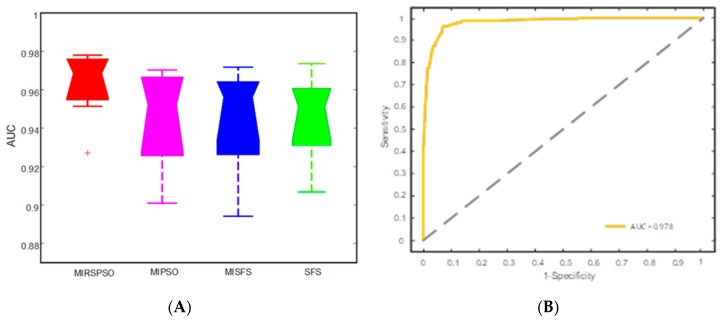
(**A**) Box-plots of the AUC for the four methods. (**B**) Receiver operating characteristic (ROC) curve of the optimal classifier. The 5-fold cross-validated ROC curve of the optimal MIRSPSO+ Support Vector Machine (SVM) classifier in the test cohort.

We used the mean values of AUC to divide the combined feature selection and classification methods into good and better performance groups (Figure 4). Combined feature selection and classification methods with AUC: 0.978 ± 0.06 (upper quartile of AUC range) are considered as highly accurate methods in Figure 4A. Figure 4B showed the confusion matrix with the MIRSPSO+RF classifier which revealed that the accuracy was 88.5%, and the sensitivity, specificity, PPV, and NPV were 92%, 85%, 86%, and 91.4%, respectively. The illustration of the Box-plots of the AUC for the four methods are described in Figure 5A and the 5-fold cross-validated ROC curve of cohort is shown in Figure 5B. As the results shown in Table 3, the prognosis of the MIRSPSO method was superior to conventional statistic methods in accuracy, sensitivity, and specificity.

## 3. Discussion

Cancer prognosis analysis is a complex and arduous task [27,28]. Statistical models based on patients’ tumor clinical characteristics provide better predictions/prognosis than that of human experts [29]. As the development of big data matures, solving clinical problems by bioinformatic methods will become prevalent. Likewise, tumor prognosis prediction based machine-learning methods could aid in clinical decision support. In this study, machine-leaning technique is applied to predict the prognosis index and to guide clinical treatment application. An improved algorithm called MIRSPSO is proposed for selecting relevant core index markers from all the prognostic indices, including age, primary tumor control, extracranial metastasis, KPS score, number of lesions, and max lesion volume. Firstly, core index markers were selected, and then compared with four existing methods based on the accuracy of classification. Four kinds of methods are used for improving the AUC of prediction performance (Figure 5). It achieved almost 97.8% of the AUC rate. In addition, we found that this machine-learning prognosis was superior to statistical method in terms of accuracy, sensitivity, and specificity (Table 4). Results from the experiment show that our proposed method can fully explore the importance of the relationship between the features of cancer patients, and obtain more effective and more stable results than other methods.

The proposed methods with machine learning and previous studies based on conventional statistical methods indicate that BSBM is a good predicting index in overall survival [22]. After obtaining the core attributes and the ranking of feature importance, we use the classical classifier to verify the classification accuracy. Figure 5 shows the performance of classification after attribute reduction by the compared methods. The tests were repeated 50 times with the same experiment parameters. As shown in Figure 5A, with the attribute reduction appropriately applied, the average AUC of classification from the data set increased slightly. The accuracy rates of MIRSPSO rose up by 1.72%, 1.29%, and 1.83% for each of SFS, MIPSO, and MISFS, respectively. The classification performance of the data set became more stable with attribute reduction. The proposed method obtained the smallest displacement between the upper and lower quartiles. The displacements were 0.026, 0.069, 0.077, and 0.067 for MIRSPSO, MIPSO, MISFS, and SFS, respectively. Thus, MIRSPSO is superior to the other three methods in terms of both the highest and the lowest values of AUC.

Qian et al. [30] showed the differentiation of glioblastoma from solitary brain metastases using radiomic machine-learning classifiers. They used machine learning to classify the glioblastoma from solitary brain metastases, which declared a novel diagnosis path for clinicians. Zhang et al. also used machine learning classifiers for prognostic biomarkers of advanced nasopharyngeal carcinoma, which showed a better performance [28]. BSBM, as a predictor of cancer prognosis index, was evaluated by the conventional statistic method and machine learning. Our proposed method can fully identify the core attributes and achieve a higher and more accurate predicting ability due to the consideration of the attribute dependence of each feature and the mutual information between attributes from cancer patients. Thus, mining prognosis via an artificial intelligence technique demonstrates a novel method to acquire the most accurate prognostic index.

Although our study has achieved better results than other methods, it still has the following limitations. Firstly, the number of cases we used were still very limited, which restricts the predictive model’s generalizability and accuracy of the power of artificial intelligence. Secondly, we only studied the prognostic indices and the relationship between prognostic indices. There are still other features to be explored in order to understand more about the relationship of the importance of all the features. Therefore, in the future, we are planning to improve the efficiency of the classification of the selected features, where more comprehensive features of cases from clinical data and more complete features of importance for mining will be studied. Meanwhile, we will combine our method with deep learning technology to deeply explore the significant relationship of cancer features. The results will be used to enhance decision making quickly and accurately to achieve better prognostic indices and enhance anticancer therapeutic effects. The small sample size, however, remains as a challenge even in the previous study [27], which needs to be overcome.

## 4. Materials and Methods

### 4.1. Patient Enrollment

For the experimentation, we obtained 700 patients’ data from a hospital between 1 January 2010 and1 December 2018. Firstly, the data downloaded from the relevant cases originated from a special hospital digital database. All the patients’ demographics are listed in Table 5. What was stored in the database are records of700 patients who underwent gamma-knife radiosurgery for brain metastases at the 986 Hospital of People’s Liberation Army Air Force, in China. The ages of the patients ranged from 16 to 92 years old, comprised of 456 (65.1%) males and 244 (34.9%) females. All patients were diagnosed by bronchoscopic biopsy, head enhanced 1.5T magnetic resonance imaging (MRI) (SIEMENS Symphony, Germany), and computer tomograph (CT). The patients’ characteristics and demographics are shown in Table 5. The hospital ethics approval was obtained for this database analysis and all patients signed the informed consent form.

### 4.2. Treatment Process

The patients were fitted with stereotactic headframes and received gamma-knife (Masep SRRS, Shenzhen, China) during radiosurgery, with MRI head enhancement scanning conducted in advance. The radiotherapy dose in the target area was prescribed according to the NCCN guideline that the maximum doses were 28Gy,25Gy,17Gy,14Gy, and 12 Gy in accordance with the maximum diameters of <10mm, 20 mm, 21–30 mm, 31–40mm, >40mm, respectively. Of the 700 patients, 197 patients had received primary tumor surgical resection before radiotherapy and only 34 patients had received molecular targeted therapy after radiotherapy.

### 4.3. Patients Survival and Prognostic Factors

At the end of the follow-up, it was found that all the 700 patients included in this study were dead and the missing patients who were not properly followed-up were excluded. The survival curve of each prognostic indices was obtained by K–M survival analysis. The RPA was defined as KPS score greater than 70, aged younger than 65, good primary tumor control, and no extracranial metastasis, median survival time was 7.1 months. Such patients were considered as having good prognosis, which was first established in 1997. Therefore, Sperduto, et al. [31] compared GPA predictive ability with RPA, SIR, and BSBM by statistical methods. Generally, Watanabe et al. [20] considered age was an prognostic factor in brain metastases patients, which was also a component of RPA, GPA, SIR, and BSBM. Then, we used four prognosis indexes RPA, GPA, SIR, and BSBM to predict metastases patient survival.

### 4.4. Feature Selection with MIRSPSO

**Figure 6:**
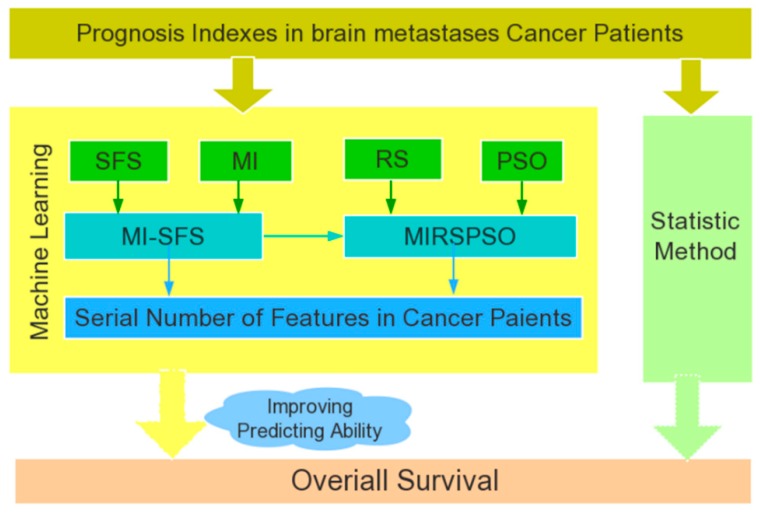
The relationship among the methods used in this study.

Mutual Information and rough set with particle swarm optimization (MIRSPSO)is a method of attribute reduction combining the binary particle swarm optimization algorithm. First, according to the principle in the binary particle swarm optimization algorithm, the solution of each dimension of each particle corresponds to either “1” or “0”, which is correspondingly expressed as the attribute of the corresponding dimension in a decision table. Through hexadecimal transformation, the values of “0” and “1” are given practical meaning. Digit 0 means the attribute in the decision table is not selected, and digit 1 means the attribute is selected. In this way, the execution mechanism can switch between particle swarm optimization algorithm and attribute reduction algorithm, embracing the power of both functions. Second, define NDT = (*U*, *C*, *D*) as the data decision information table, where *U* is the domain, *C* is the condition attribute, and *D* is the decision attribute. The mutual information is used as the fitness function and the termination condition of the loop is set to the maximum number of iterations [32]. Thirdly, the global optimal solution [33] of the population in the search space is obtained by iterative optimization; the search agents are coded as the attribute condition selection results based on mutual information and the attribute reduction theory. Finally, a minimum subset of attributes which are reduced from the full feature set is retained in the decision information table. The resultant feature subset satisfies the optimization conditions and they are optimal. Figure 6 illustrates the relationship among the computational methods used in this study.

### 4.5. Feature Classification Methods

We applied seven supervised machine-learning algorithms including K-nearest neighbor (KNN), Backpropagation (BP) Neural Network, decision tree (DT), logistic regression (LR), Random forest (RF), Naive Bayes (NB), and Support Vector Machine (SVM) [30]. Feature classification methods were all implemented using the MATLAB (version 2018a) machine-learning library tool kit, which provides an overall and good user interface to accesses many machine-learning algorithms. Classifiers were trained using 10-fold cross-validation method in the training cohort, and their prognostic performance was then evaluated in the validation cohort using the area (AUC) under the receiver operator characteristic (ROC) curve.

### 4.6. Identification of Excellent Performance Groups

We used the mean values of AUC to divide the combined feature selection and classification methods into good and excellent performance groups. Combined feature selection and classification methods with AUC are considered as highly accurate methods.

### 4.7. Statistical Analysis

All data were assessed by the Student’s t-test or chi-square test, as appropriate. A threshold 0.001 was set as a two-tailed statistical significance level. The statistical analysis and figure plots were performed using GraphPad software (Prism 8 version, San Diego, CA, USA).

## 5. Conclusions

In this study, an improved innovative algorithm method (MIRSPSO) was established to select the corresponding core index marker from all prognostic indices regarding brain metastases cancer patients. It may provide a feasible and convenient method to seek optimized index markers for clinical use.

## Figures and Tables

**Table 1:** The description (letter representation, name) of cancer features in patients.

Cancer Feature	Label
Age	A
Karnofsky Performance Status	K
Extracranial metastasis	E
Primary tumor control	P
Number of lesions	N
Max lesion volume	M
If chemical therapy	C

**Table 2:** Importance ranking of cancer features in patients by different methods.

No.	Methods	Cancer Features in Patients						
1	SFS	C	A	E	K	P	M	N
2	MISFS	A	P	E	K	C	N	M
3	MIPSO	P	E	A	C	K	N	M
4	MIRSPSO	P	C	A	E	N	K	M
		P	A	C	E	N	K	M
		P	E	C	A	N	K	M
		P	A	E	C	K	N	M
		P	A	E	C	K	N	M
		A	P	C	E	K	N	M
		P	E	A	C	N	K	M
		P	A	E	C	N	K	M
		P	A	E	C	N	K	M

SFS: Sequential Feature Selection; MISFS: Sequential Feature Selection with mutual information; MIPSO: mutual information with particle swarm optimization; MIRSPSO: mutual information and rough set with particle swarm optimization.

**Table 3:** Comparison of importance ranking of cancer features in patients for different weights in MIRSPSO.

No.	Cancer Features in Patients							$\omega_{1}\;\mathrm{Weight}\;(1)$	$\omega_{2}\;\mathrm{Weight}\;(2)$
1	P	C	A	E	N	K	M	0.9	0.1
	0.9074	0.8535	0.7286	0.6303	0.4771	0.3896	0.3247		
2	P	A	C	E	N	K	M	0.8	0.2
	0.9278	0.8634	0.8021	0.6935	0.455	0.4011	0.3546		
3	P	A	E	C	N	K	M	0.7	0.3
	0.9322	0.8734	0.7559	0.6765	0.4834	0.3724	0.3618		
4	P	A	E	C	K	N	M	0.6	0.4
	0.8921	0.8105	0.765	0.6267	0.4933	0.4	0.3911		
5	P	A	E	C	K	N	M	0.5	0.5
	0.9213	0.7705	0.7068	0.703	0.4267	0.4201	0.3692		
6	A	P	C	E	K	N	M	0.4	0.6
	0.7183	0.6437	0.5902	0.5559	0.4757	0.43	0.3783		
7	P	E	A	C	N	K	M	0.3	0.7
	0.891	0.9211	0.7948	0.7774	0.4469	0.351	0.3		
8	P	A	E	C	N	K	M	0.2	0.8
	0.9335	0.91	0.8283	0.7632	0.4528	0.3439	0.3267		
9	P	A	E	C	N	K	M	0.1	0.9
	0.9546	0.8973	0.7751	0.6748	0.4486	0.3382	0.36		

**Table 4:** Comparison of predictive performance between machine-learning and statistic methods in the test cohort.

Statistic Method	RPA	GPA	SIR	BSBM	Overall Survival	Machine Learning
MST	19	26	25	23	24	-
Sensitivity	0.67	0.71	0.59	0.88	-	0.92
Specificity	0.39	0.33	0.43	0.46	-	0.85
Accuracy	0.682	0.655	0.611	0.758	-	0.885
PPV	-	-	-	-	0.83	0.86
NPV	-	-	-	-	0.65	0.914
P	<0.0001 ^a^	<0.0001 ^a^	<0.0001 ^a^	<0.000 ^a^	-	-

^a^ Chi-square test. Abbreviations: MST, medium survival time (months); PPV, positive prediction value; NPV, negative prediction value.

**Table 5:** The characteristics and demographics of the patients.

Characteristics		N (%)
Patients		700
Gender	Male	456 (65.1%)
	Female	244 (34.9%)
Age(years)	Median	55
	Range	48 (16–92)
KPS	Median	75
	Range	30 (55–95)
Primary tumor type	NSCLC	635 (90.7%)
	Breast cancer	57 (8.1%)
	Other	8 (1.2%)
Primary tumor control	No	319 (45.6%)
	Yes	381 (54.4%)
Number of lesions	Median	3
	Range	5 (1–6)
Tumor volume(mL)	Median	6
	Range	40.4 (0.04–49)
Maximum diameter(mm)	<10	28 (4%)
	10–20	189 (27%)
	21–30	245 (35%)
	31–40	205 (29.3%)
	>40	33 (4.7%)
Type of therapy	SRS	225 (32.1%)
	Fractionated SRS WBRT	127 (18.1%)
	SBRT	133 (19%)
	Surgical resection	197 (28.1%)
Extracranial metastasis	No	470 (67.1%)
	Yes	230 (33.9%)
Histology classification	Adenocarcinoma	170 (24.3%)
	Squamous cell carcinoma	120 (17.1%)
	Large cell carcinoma	87 (12.4%)
	In situ carcinoma	195 (27.9%)
	Invasive carcinoma	128 (18.3%)
Molecular classification	EGFR	23 (3.3%)
	KRAS	17 (2.4%)
	BRAF	4 (0.6%)
	TP53	9 (1.3%)
	ALK	15 (2.1%)
	TNBC	8 (1.1%)
	HER2	9 (1.3%)
	PTEN	7 (1.0%)
Pattern of dissemination	Blood	361 (51.6%)
	Lymph	255 (36.4%)
	Others	84 (12%)

NSCLC: Non-small Cell Lung Cancer; SRS: Stereotactic Radiosurgery; WBRT: Whole Brain Radiotherapy; SBRT: Stereotactic Body Radiation Therapy; EGFR: Epidermal Growth Factor Receptor; KRAS: K-Ras gene; BRAF: B-Raf gene; TP53: TP53 gene; ALK: Anaplastic lymphoma kinase; TNBC: Triple-negative breast cance; HER2: human epidermal growth factor receptor 2; PTEN: Phosphatase and tensin homolog.

## Abbreviations

PI	Prognostic Index
BM	Brain Metastases
NSCLC	Non-small Cell Lung Cancer
RPA	Recursive Partitioning Analysis
SIR	Score Index for Radiosurgery
GPA	Graded Prognostic Assessment
BSBM	Basic Score for Brain Metastases
AUC	Area under the receiver operating characteristic curve
SD	Standard deviation
LR	Logistic Regression
SVM	Support Vector Machine
RF	Random Forest
DC	Distance Correlation
MIRSPSO	Mutual Information and Rough set with Particle Swarm Optimization
NB	Naive Bayes
MST	Median Survival Time
WBRT	Whole Brain Radiotherapy
SRS	Stereotactic Radiosurgery
MRI	Magnetic Resonance Imaging
OS	Overall Survival
K-M	Kaplan-Meier
KPS	Karnofsky Performance Status
